# *Helicobacter pylori* promotes gastric intestinal metaplasia through activation of IRF3-mediated kynurenine pathway

**DOI:** 10.1186/s12964-023-01162-9

**Published:** 2023-06-16

**Authors:** Xinhua Liang, Wenjun Du, Ling Huang, Li Xiang, Wenxu Pan, Fangying Yang, Fengfeng Zheng, Yongwu Xie, Lanlan Geng, Sitang Gong, Wanfu Xu

**Affiliations:** 1 Department of Gastroenterology, Guangzhou Women and Children’s Medical Center, Guangdong Provincial Clinical Research Center for Child Health, Guangzhou Medical University, Guangzhou, 510623 China; 2 Guangzhou Women and Children’s Medical Center, Guangdong Provincial Clinical Research Center for Child Health, Guangzhou Institute of Pediatrics, Guangzhou Medical University, Guangzhou, 510623 China; 3 Department of Infectious Diseases, The Affiliated Hospital of Putian University, Putian, 351100 China; 4 Department of Hematology, Zhuhai Center for Maternal and Child Health Care, Zhuhai, China

**Keywords:** *H. pylori*, IRF3, Kynurenine pathway, Xanthurenic acid, Gastric intestinal metaplasia

## Abstract

**Background:**

Metabolic reprogramming is a critical event for cell fate and function, making it an attractive target for clinical therapy. The function of metabolic reprogramming in Helicobacter pylori (*H. pylori*)-infected gastric intestinal metaplasia remained to be identified.

**Methods:**

Xanthurenic acid (XA) was measured in gastric cancer cells treated with *H. pylori* or *H. pylori* virulence factor, respectively, and qPCR and WB were performed to detect CDX2 and key metabolic enzymes expression. A subcellular fractionation approach, luciferase and ChIP combined with immunofluorescence were applied to reveal the mechanism underlying *H. pylori* mediated kynurenine pathway in intestinal metaplasia in vivo and in vitro.

**Results:**

Herein, we, for the first time, demonstrated that *H. pylori* contributed to gastric intestinal metaplasia characterized by enhanced Caudal-related homeobox transcription factor-2 (CDX2) and mucin2 (MUC2) expression, which was attributed to activation of kynurenine pathway. *H. pylori* promoted kynurenine aminotransferase II (KAT2)-mediated kynurenine pathway of tryptophan metabolism, leading to XA production, which further induced CDX2 expression in gastric epithelial cells. Mechanically, *H. pylori* activated cyclic guanylate adenylate synthase (cGAS)-interferon regulatory factor 3 (IRF3) pathway in gastric epithelial cells, leading to enhance IRF3 nuclear translocation and the binding of IRF3 to KAT2 promoter. Inhibition of KAT2 could significantly reverse the effect of *H. pylori* on CDX2 expression. Also, the rescue phenomenon was observed in gastric epithelial cells treated with *H. pylori* after IRF3 inhibition in vitro and in vivo. Most importantly, phospho-IRF3 was confirmed to be a clinical positive relationship with CDX2.

**Conclusion:**

These finding suggested *H. pylori* contributed to gastric intestinal metaplasia through KAT2-mediated kynurenine pathway of tryptophan metabolism via cGAS-IRF3 signaling, targeting the kynurenine pathway could be a promising strategy to prevent gastric intestinal metaplasia caused by *H. pylori* infection.

Video Abstract

**Supplementary Information:**

The online version contains supplementary material available at 10.1186/s12964-023-01162-9.

## Background

Helicobacter pylori (*H. pylori*), classified as a class I carcinogen by World Health Organization, is a critical factor for gastritis and gastric intestinal metaplasia (IM) [1, 2]. IM has been regarded as a precancerous lesion in the *H.pylori*-induced metaplasia-dysplasia-carcinoma sequence, which was characterized by increased CDX2 and/or MUC2 expression [3, 4], an intestinal specific homeobox gene that regulated the development and maintenance of the intestinal mucosa phenotype [5]. The previous work has revealed that *H. pylori* virulence factor CagA contributed to gastric carcinoma cells invasiveness through upregulating CDX2-mediated Claudin-2 expression in AGS gastric cancer cells, thereby disrupting tight junction [6], and *H. pylori* promoted CDX2-mediated IM through NF-κB [7], SOX2 [8] and Activin A receptor type I (ACVR1) [3]. However, the mechanisms that regulate CDX2 expression during *H. pylori*-induced IM have not been fully defined.

Alteration of metabolic reprogramming plays a crucial role in the development of *H. pylori*-related gastric disease [9, 10]. For instance, in H.pylori-infected macrophages, cystathionine γ-lyase (CTH, also known as CSE) triggered the mammalian reverse transsulfuration pathway (RTP)-mediated SAM metabolism and induced macrophages activation through enhancing mitochondrial function and glycolysis [11], and *H.pylori* aggravated the severity of gastric inflammation and the development of premalignant lesions in the setting of iron deficiency through enhanced bile acid production [12, 13]. What’s more, the study showed that *H. pylori* virulence factor CagA contributed to 5-Fu resistance of gastric cancer cells through upregulating Hexokinase 2 (HK2) and LDHA-mediated glycolysis [14]. In addition, our previous work have demonstrated that fatty acid synthase (FASN) and ATP citrate lyase (ACLY)-mediated De novo lipogenesis (DNL) was increased in gastric mucosa with *H.pylori* infection [15], and HK2-mediated glycolysis was enhanced in gastric mucosa of patients with *H.pylori* infection [16]. Interestingly, bile acids (BAs) was reported to induce CDX2-mediated gastric intestinal metaplasia through HNF4α [17] and a major RNA N6-adenosine demethylase, named alkylation repair homolog protein 5 (ALKBH5) [18] as well as FXR [19, 20]. However, the further work was required to elucidate the role of metabolism reprogramming during *H. pylori* infection.

Kynurenine pathway is the main route of tryptophan metabolism and produces several intermediate metabolites with various biologic properties [21, 22]. Tryptophan(Trp) could be converted into kynurenine(KYN) by the first and rate-limiting enzymes indoleamine 2,3-dioxygenase (IDO) and/or tryptophan 2,3-dioxygenase (TDO) [23]. KYN could be catalyzed into 3-Hydroxy kynurenine (3HK) by kynurenine 3-monoxygenase (KMO), which can be further metabolized into 3-Hydroxy anthranilic acid (3HAA) and xanthurenic acid (XA) by kynureninase (KYNU) and kynurenine aminotransferase II (KAT2), respectively [24, 25]. What’s more, 3HAA could be further converted into quinolinic acid (QUIN) and picolinic acid (PIC) by 3-hydroxyanthranilate-3,4-dioxygenase (QPRT) and 7, 2-amino-3-carboxymuconic acid semialdehyde decarboxylase (ACMSD), respectively. In addition, kynurenine can be converted to anthranilic acid (QA) [26]. Recently, Trp depletion and KYN metabolites generation were identified in plasma of GC through disease progression within the Correa’s cascade [27], and enhanced KYN level in serum has been showed to facilitate Treg induction in vitro to promote chemotherapy resistance in gastric cancer cells through IL-10/STAT3/BCL2 [28]. What’s more, KYN interacted with AHR to suppress immune and subsequently, susceptibility to gastric cancer [29]. In addition, L-Kyn was confirmed to promote COL12A1 expression to induce gastric cancer cell growth and migration [30]. Importantly, the clinical data further showed significant increased kyn/Trp ratio, observed in *H. pylori* seropositive cancer patients, could be identified as potential gastric cancer biomarkers (<10.1515_pteridines.2010.21.1.110.pdf>) [31]. Herein, in this work, we further demonstrated that *H. pylori* triggered IRF3-mediated kynurenine pathway, leading to XA production, which induced CDX2 expression in gastric epithelial cells. Inhibition of KAT2 expression by PF-04859989 hydrochloride could reverse the promotion of CagA and VacA on CDX2 expression. Mechanically, *H. pylori* triggered cGAS/STING/IRF3 pathway to enhance IRF3 nuclear translocation and increase the binding of IRF3 to KAT2 promoter confirmed by luciferase and ChIP assay in BGC823 cells. Inhibition of IRF3 in gastric epithelial cells could rescue KAT2 expression induced by *H. pylori *in vivo and in vitro. Taken together, we identified a previously unreported mechanism that *H. pylori*-mediated gastric IM through activation of kynurenine pathway.

## Methods and materials

### Reagents and antibodies

#### Reagents

Dulbecco’s modified Eagle’s medium (DMEM, C11995500BT), and fetal bovine serum (FBS, 10099141C) were purchased from Life Technologies (Carlsbad, CA, USA); Recombinant helicobacter pylori CagA (ab224836), VacA (ab225655), Phenylmethanesulfonyl fluoride (PMSF, P0100) and protease inhibitor cocktail (PIC, P6730) were from Solarbio (Beijing, China); Ratio-immunoprecipitation assay (RIPA) lysis buffer(P0013B), Nuclear and Cytoplasmic Protein Extraction Kit (P0028), bicinchoninic acid (BCA) protein assay kit (P0012) and Beyozol RNA Isolation Kit (R0011) were from Beyotime Biotechnology (Shanghai, China); All-in-One First-Strand cDNA Synthesis Kit (QP006) and All-in-One qPCR Mix (QP005) were obtained from GeneCopoeia (Rockville, MD, USA). Dual-Luciferase Reporter Assay was purchased from Promega. SimpleChIP® Plus Enzymatic Chromatin IP Kit (Magnetic Beads) was purchased from CST. PF-04859989 HCI(T28368), MRT67307 HCI(1190378–57-4(free base))( T5162) and Xanthurenic Acid(S4774) was from Topscience and Selleck, respectively. All ultrapure reagents were from Sigma (St Louis, MO, USA).

#### Antibodies

Antibody against CDX2(60243–1-Ig, Proteintech1:2000 for WB, 1:800 for IF of human sample), CDX2 Monoclonal Antibody(14H6) (YM3057, immunoway, 1:800 for IF and IHC of mice gastric mucosa), MUC2(ab272692, Abcam,1:2000 for WB, 1:800 for IF), TDO2(15880–1-AP, Proteintech, 1:1500 for WB), KMO(YT6625, immunoway, 1:2000 for WB), AADAT (A13089, Abclonal, 1:1000 for WB), AADAT polyclonal antibody(13031–1-AP, Proteintech,1:800 for IHC/IF), α-tubulin(RM2007, Ray, 1:4000 for WB), Histone 3 (RM2005, Ray, 1:4000 for WB), phospho-cGAS-S291 Rabbit pAb(Abclonal, AP1176, 1:1000 for WB), cGAS Rabbit pAb (Abclonal, A8335, 1:1000 for WB), phospho-STING-S365 Rabbit pAb (Abclonal, AP1199, 1:1000 for WB), STING/TMEM173 Rabbit pAb (Abclonal, AP1176, 1:1000 for WB); IRF3(Immunoway, YT2398, 1:1000 for WB), Phospho-IRF3(s396)(Immunoway,YP0326, 1:1000 for WB), phospho-IRF3 (Ser396) Antibody (AF2436, affinity, 1:800 for IF), alexa-488- and 594-conjugated secondary antibodies were from Immunoway (Beijing, China).

### Cell culture, treatment, and transfection

As described in our pervious study [32], GES-1 and BGC823 cells were from American Type Culture Collection and were cultured in DMEM supplemented with 10% fetal bovine serum (FBS) according to the manufacturer’s recommendations. For treatment, gastric epithelial cells were used in the whole study and treated with CagA(1ug/ml), VacA(1ug/ml) or CagA combined with vacA for 48 h. For transfection, plasmid or siRNA was transfected into cell using lipofectamine 3000 according to the manufacturer’s protocol. siRNA targeted IRF3(sc-35710) was purchased from Santacruz. KAT2 plasmid was from Youbio (Changshang, China).

### *H. pylori bacterial *strains culture and infection

According to the work from Soutto et al. [33], *The CagA*^+^ *H. pylori* strains “J166”, a clinical isolate of human-derived *H. pylori, was* cultured on columbia agar plates containing 5% (v/v) sheep blood at 37 °C under microaerobic conditions. *H. pylori* was collected and resuspended in PBS. For infection, BGC823 (a human gastric carcinoma undifferentiated cell line) was cultured and washed with PBS three times and then infected with *H. pylori* at an MOI of 100 for 48 h after analysis of the quantity of bacteria by spectrophotometry.

### RNA isolation and quantitative real-time PCR

As described in our pervious study [34], Total RNA was extracted and reverse transcripted into cDNA according to Beyozol RNA Isolation Kit and the All-in-one™ first-strand cDNA synthesis kit (Genecopoeia™, FulenGen), respectively. Quantitative PCR (qPCR) were carried out using the All-in-one™ qPCR mix (Genecopoeia™, FulenGen) according to the manufacturer’s instructions. Primer sequences used in this study were as followed: CDX2: forward:5′-CTCGGCAGCCAAGTGAAAACCA-3′, and reverse: 5′-GCTTTCCTCCGGATGGTGATGTA-3′ [35]; Villin: forward:5′-TCGGCCTCCAGTATGTAG-3′, and reverse: 5′- CGTCTTCGGGGTAGAACT-3′ [36]; MUC2: forward:5’-CAGGATGGCGCCTTCTGCTA-3’, and reverse:5’-ATGCTGCTCCAAGCTGAGGT-3’ [37]; TDO2: forward:5’-TCCTCAGGCTATCACTACCTGC-3’ and reverse: 5’-ATCTTCGGTATCCAGTGTCGG-3’; KMO: forward:5’-TGCCATCCCTCTAATTGGAGA-3’ and reverse: 5’-GCCCGCATTCATTCCTTGC-3’; KAT2: forward:5’- CACTTCAGTATTCTCCGAGTGC-3’ and reverse: 5’- AGCAGGTTCATCTAGGAGGAC-3’; UBC (internal control): forward:5’-ATTTGGGTCGCGGTTCTTG-3’ and reverse: 5’-TGCCTTGACATTCTCGATGGT-3’.

### Western blotting (WB) analysis

As described in our work [5], after treatment, cells were collected and lysed in RIPA buffer containing 1% protease inhibitors, SDS-PAGE was performed to separate after quantitation. Proteins were transferred nitrocellulose (NC) membranes (PALL) and incubated primary antibodies for overnight at 4 °C. the further incubation with the secondary antibodies for 1 h at room temperature was required after washing with PBS. The protein level was detected using an enhanced chemiluminescence (Perkin Elmer).

### Immunofluorescence (IF) analysis

As described in pervious study [38], after treatment, fixed and permeabilized gastric epithelial cell or gastric mucosa tissue slides were incubated with 2% BSA in PBS/0.05% Triton X-100 for 30 min. the slips were incubated with the primary antibody overnight at 4 °C, followed by incubation with Alexa-488- or Alexa 594- conjugated secondary antibodies for 1 h at room temperature. The coverslips were mounted onto glass slides with Prolong Gold Antifade reagent (after staining the nuclei with DAPI), and stained cells were imaged with a Leica TCS SP8 Inverted Fluorescence Microscope (Leica Microsystems). Post-acquisition processing (brightness, opacity, contrast, and color balance) was applied to the entire image and accurately reflected the results of the original image. For statistical analysis, as described in our previous work [39], 5 areas of each single gastric mucosa section were randomly selected for quantification. The number per square millimeter was counted and analyzed using Leica X image analysis software (Leica, Hamburg, Germany) and ImageJ software (National Institutes of Health, MD, USA).

### Immunohistochemical staining

Briefly, the paraffin-embedded tissues were deparaffinized in xylene and gradient ethanol. Three percent hydrogen peroxide (H_2_O_2_) solution was used to block endogenous peroxidase activity after antigen retrieval with sodium citrate buffer. The slide was incubated overnight at 4 °C with the primary antibody. After washing with PBS, the sections were incubated with secondary antibody using PV-6001/6002 kit (Zhongshan Biotech, Beijing, China). The slides were stained with diaminobenzidine (DAB) chromogen and counterstained with hematoxylin. All images were acquired under a microscope (Leica, Hamburg, Germany). The immunoreactivity of the samples was scored for intensity (scaled 0–4) and frequency (scaled 0–4). For the statistical analysis, the intensity and frequency of targets were transformed into a composite expression score using the following formula intensity × frequency. The score ranges from 0 to 16.

### Xanthurenic acid (XA) detection assay

After serum starved for 24 h, cells were treated with CagA and VacA or CagA combined with VacA or *H. pylori*, respectively, for 48 h, the level of XA in indicated supernatant were collected and detected according to the manufacturer’s protocol (MEIMIAN, MM-9263001).

### Luciferase reporter assay

Cells were co‐transfected with the promoter reporter plasmid (KAT2‐Luc) or internal control plasmid (pGL4.74[hRluc/TK], Promega) with siRNA targeted IRF3. Twenty‐four hours after transfection, the cells were treated for further 24 h, The Firefly and Renilla luciferase value in cells were measured using the Dual‐Luciferase Reporter Assay System (Promega).

### Chromatin immunoprecipitation (ChIP)

As described in previous studies [34, 40], gastric epithelial cells were grown up to 80% confluence, the cell was treated with serum-free medium for 24 h, and stimulated for another 1 h, the ChIP was performed using the SimpleChIP® Plus Enzymatic Chromatin IP Kit (Magnetic Beads) according to the manufacturer’s protocol. Quantitative PCR of co-immunoprecipitated genomic DNA fragments was performed with specific primers was synthesized from thermo life. Primers to amplify the proximal region of the KAT2 promoter containing a IRF3‐binding site was forward:5’- TGCAGAAAAGTTGAGGGAGGT-3’ and reverse: 5’- TTTTTGGGTCTAGCACCTTGCC-3’.

### Animals infection model

As described in Soutto et al. [33], for in vivo, 6–8 weeks of age were orally gavaged with rodent-adapted CagA^+^ *H. pylori* strain pre-murine Sydney Strain 1 (PMSS1, 2 × 10^9^ CFU)/mouse) every other day or challenged with Brucella Broth (BB) as an uninfected control group, After two weeks, one group of PMSS1 infected mice received PF-04859989(50 mg/kg) or MRT67307 (50 mg/kg) through intraperitoneal injection once every 2 days. Uninfected and H. pylori infected mice were used as control groups receiving injection with 1xPBS. Mice were euthanized a day after the last injection. All mice were euthanized to collect gastric mucosa for experiment.

### Ethical approval of clinical sample studies

This study was conducted in a cohort of 14 Hp-infected patients diagnosed with gastric intestinal metaplasia, 12 healthy control upon the declaration of Helsinki as reflected in a prior approval by the institution's human research committee from 2016 to 2022 approved by the affiliated hospital of Putian university. Written informed consent was given by the caregiver of the patient for his clinical records used, which are not publicly available since the database is currently not anonymous and contains all patient's name; however, it could be available upon request.

### Statistics analysis

GraphPad Prism V software was used to conduct all analysis. A *P* value less than 0.05 was considered to be statistical significant. Statistical differences among groups were determined by Student's t test, one sample t tests, one‐way ANOVA or two‐way ANOVA were used to determine the significance.

## Results

### KAT2/XA promoted gastric intestinal metaplasia

**Fig. 1:**
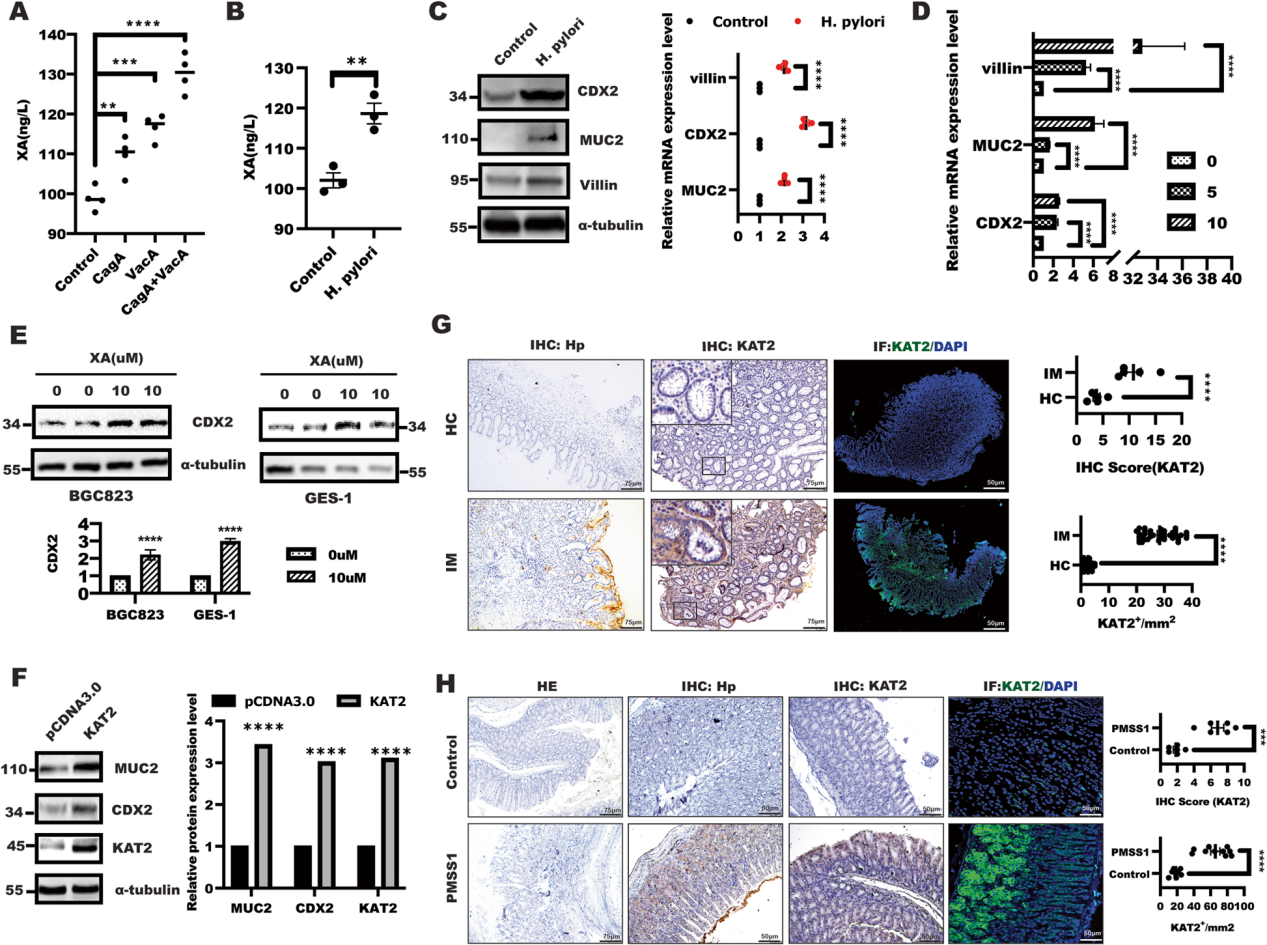
Enhanced XA/KAT2 in *H. pylori infection* induced gastric epithelial. Elisa was employed to detect the level of xanthurenic acid (XA) in BGC823 cells in response to CagA, VacA and CagA + VacA (**A**) or *H. pylori* infection (**B**) for 48 h, data presented as the means ± s.d. of three independent experiments and were analyzed by one-way ANOVA with multiple comparisons, followed by Dunnett post hoc test for significance, **p < 0.01, ***p < 0.001.****p < 0.0001. **C** After serum starvation for 24 h, BGC823 cells were incubated with *H. pylori* for additional 48 h. The total lysate was collected to separate by SDS to detect indicated protein expression. The band intensity was measured and quantified by Image J. Data represent the mean ± s.d. of three independent experiments and were analyzed by t test for significance. ****p < 0.0001. **D** the total RNA was isolated and quantitative real-time PCR was performed to analyzed indicated gene expression in BGC823 cells treated with XA at 5uM and 10uM. Data presented as the means ± s.d. of three independent experiments and were analyzed by one-way ANOVA with multiple comparisons, followed by Dunnett post hoc test for significance, ****p < 0.0001. **E** immunoblotting was used to detect CDX2 expression in BGC823 and GES-1 cells treated with 10uM XA for 48 h, respectively. α-tubulin was determined to ensure equal loading. Data presented as the means ± s.d. of three independent experiments and were analyzed by t test for significance, ****p < 0.0001. **F** BGC823 cells were transfected with pCDNA3.0 and KAT2 plasmid for 48 h, and WB was used to test indicated protein expression, the band intensity was quantified by Image J. Data represent the mean ± s.d. of three independent experiments and were analyzed by t test for significance. ****p < 0.0001. **G-H** HE, IHC and IF assay was used to detect KAT2 expression in gastric mucosa of patients with IM and in animal model. The KAT2 expression level was quantified and analyzed by t test for significance, ***p < 0.001, ****p < 0.0001

Metabolic reprogramming is critical for determination of cell fate. Specifically, a total of 37 differentially expressed metabolites (DEMs) metabolites, including xanthurenic acid (XA), were identified in BGC823 cells treated with *H. pylori* virulence factor CagA and VacA compared with Control group by liquid chromatography-tandem mass spectrometry (LC–MS/MS) (data unpublished). Consistently, enhanced xanthurenic acid (XA), a production of kynurenine pathway, was found in BGC823 cells in response to CagA, VacA, and CagA + VacA treatment comparing with Control group confirmed by Elisa (Fig. 1A). In line with this, the increased XA level, enhanced protein and mRNA level of CDX2, MUC2 and villin expression were significantly observed in BGC823 cells in response to *H. pylori* infection (Fig. 1B and C). these results implied *H. pylori* infection induced gastric intestinal metaplasia accompanying with XA production.

To explore whether XA caused by *H. pylori* infection involved in gastric intestinal metaplasia, real-time PCR and WB analysis were utilized to examine. The results showed that XA treatment in BGC823 cells led to a significant upregulation of CDX2, MUC2 and Villin mRNA expression in a dose-dependent manner (Fig. 1D), and WB and quantified results showed that 10uM XA could induce CDX2 expression in BGC823 and GES-1 cells, respectively (Fig. 1E). Because of XA synthesis is dependent on the activity of mitochondrial KAT2, which focus us to determine the role of KAT2 in gastric intestinal metaplasia. As shown in Fig. 1F, ectopic expression of KAT2 in BGC823 cells led to enhance CDX2 and MUC2 expression. Interestingly, immunohistochemical staining and fluorescence displayed KAT2 expression was drastically increased in patients diagnosed with gastric intestinal metaplasia and positive *H. pylori* infection (IM) in comparsion with healthy control (HC) (Fig. 1G). What’s more, histological examination of the gastric mucosa revealed that mice infected with the *H. pylori* PMSS1 strain showed KAT2 expression was notably enhanced in gastric mucosa of mice with *H. pylori* PMSS1 strain infection (Fig. 1H). These findings suggested KAT2/XA was increased in response to *H. pylori* infection and promoted gastric intestinal metaplasia.

### *H. pylori* contributed to gastric intestinal metaplasia through KAT2-mediated kynurenine pathway

**Fig. 2:**
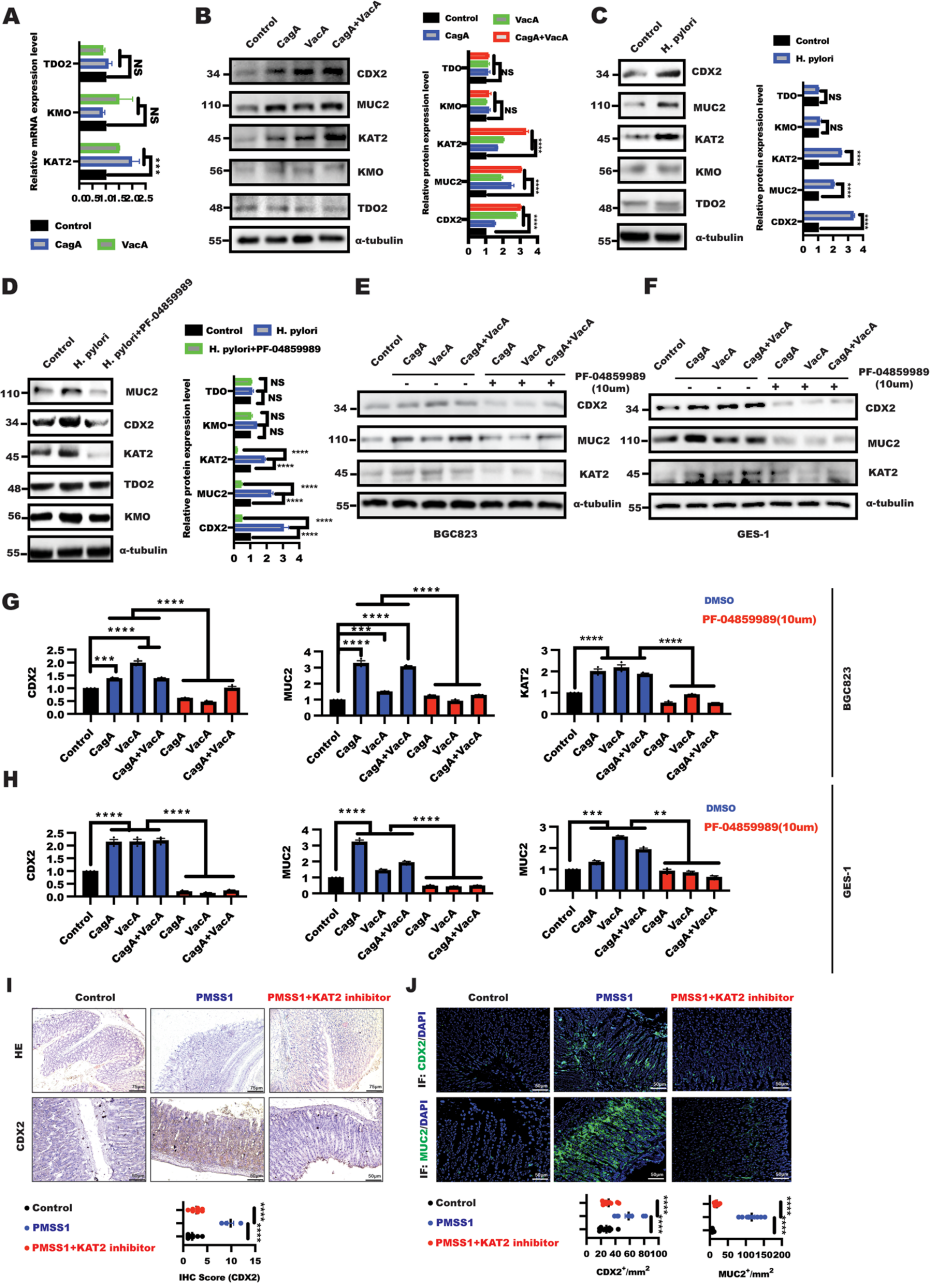
*H.pylori* triggered KAT2-mediated kynurenine pathway to induce CDX2 expression in gastric epithelial cells. **A** Real-time PCR was used to analyze the indicated genes expression in response to CagA, VacA treatment for 48 h in BGC823 and GES-1 cells, respectively. Data presented as the means ± s.d. of three independent experiments and were analyzed by one-way ANOVA with multiple comparisons, followed by Dunnett post hoc test for significance, ***p < 0.001, NS:no significant. **B** WB was performed to detect the indicated proteins expression in BGC823 cells stimulated by CagA, VacA and CagA + VacA for 48 h, α-tubulin was taken as internal control. Data presented as the means ± s.d. of three independent experiments and were quantified by two-way ANOVA with multiple comparisons, followed by Dunnett post hoc test for significance, ****p < 0.001, NS: no significant. **C** WB was employed to detect the indicated proteins expression in BGC823 cells treated with or without *H.pylori* for 48 h, α-tubulin was taken as internal control. Data presented as the means ± s.d. of three independent experiments, the band intensity was quantified and quantified by t test for significance, ****p < 0.001, NS: no significant, *n* = 3. After serum starvation for 24 h, BGC823 cells were pre-treated for 1 h with KAT2 inhibitor PF-04859989 (10uM) following by treatment as indicated (**D-F**) for additional 48 h. The total lysate was harvested to detect indicated proteins, (**D** right panel, **G-H**) Data represent the mean ± s.d. of three independent experiments and were analyzed by One-way ANOVA test for significance for D and two-way ANOVA with multiple comparisons, followed by Dunnett post hoc test for significance for E–F. ***p < 0.001, ****p < 0.0001. **I** representative histological image of gastric mucosa of C57BL/6 mice in different groups (scale bars: 75 μm), IHC staining showing CDX2 expression in gastric tissue from the indicated groups of mice (scale bars: 50 μm), Data represented the mean ± s.d. and was analyzed by One-way ANOVA test for significance. ****P < 0.0001. **J** Immunofluorescence showing CDX2 and MUC2 expression in gastric mucosa tissue in indicated group, respectively. Data represented the mean ± s.d. and was analyzed by One-way ANOVA test for significance. ****P < 0.0001

The above results implied that kynurenine pathway of tryptophan metabolism might play a vital role in *H. pylori*-induced gastric intestinal metaplasia, which focused us to explore the effect of *H. pylori* on the rate-limiting enzymes expression of 3-HK branch of tryptophan metabolism, including TDO2, KMO and KAT2. Our results demonstrated that treatment with *H. pylori* virulence factor CagA/VacA in BGC823 cells led to a significant upregulation of KAT2 expression at the mRNA level compared with control group; no significant difference in the expression of KMO and TDO2 was observed (Fig. 2A). In line with this, the result from WB and quantified analysis showed that *H. pylori* virulence factors, including CagA and VacA, or *H. pylori* infection resulted in enhancing CDX2, MUC2 and KAT2 expression in BGC823 cells, while failed to alter KMO and TDO2 expression (Fig. 2B and C). Taken together, these results suggested *H. pylori* promoted kynurenine pathway by triggering KAT2 expression, leading to XA production.

To further confirm whether KAT2 was required for in the *H. pylori*-mediated gastric intestinal metaplasia, the rescue experiment was performed through KAT2 inhibitor PF-04859989 [41, 42]. As shown in Fig. 2D, KAT2 inhibition in BGC823 cells drastically overcame the effect of *H. pylori* on gastric intestinal metaplasia characterized by enhanced CDX2 and MUC2 expression. What’s more, inhibition of KAT2 by PF-04859989 in BGC823 and GES-1 cells could largely abrogate the upregulation of CDX2 and MUC2 expression caused by *H. pylori* virulence factors (Fig. 2E-H). Most importantly, In vivo results also further confirmed that PF-04859989 alleviated the gastric intestinal metaplasia caused by *H. pylori* PMSS1 infection as confirmed by CDX2 expression through IHC and IF (Fig. 2I), in addition to IHC, IF also showed reduced CDX2 and MUC2 expression level was observed in *H. pylori* PMSS1 infection receiving KAT2 inhibitor treatment (Fig. 2J). Taken together, these findings suggested that *H. pylori* induced gastric intestinal metaplasia through KAT2-mediated kynurenine.

### IRF3 was required for *H. pylori*-mediated KAT2 expression

**Fig. 3:**
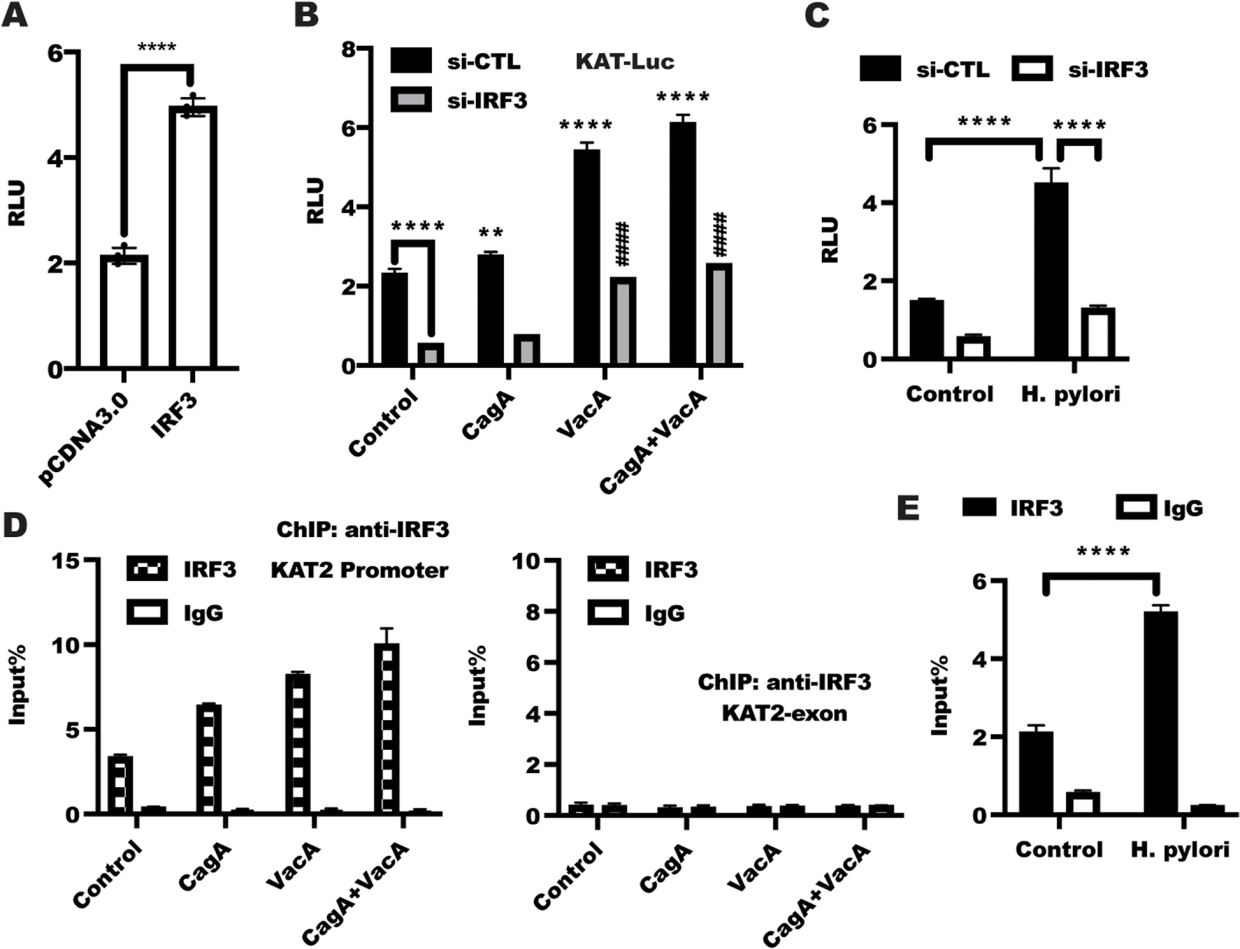
IRF3 was crucial for *H. pylori*-mediated KAT2 transactivation. **A** BGC823 cells were co-transfected with indicated plasmids combined with KAT2-Luc plasmid and a control Renilla luciferase expression vector for 48 h, the relative luciferase unit (RLU) was presented as the fold activation relative to Renilla luciferase activity. Data presented as the means ± s.d. of three independent experiments and was analyzed by t test, ****p < 0.0001. **B** After transfection with si-CTL and si-IRF3 combined with KAT2-Luc and Renilla plasmids, respectively, for 24 h, BGC823 cells were starved for 12 h following by stimulation with CagA, VacA and CagA + VacA for further 48 h, the RLU was detected using dual‐luciferase reporter assay system (Promega). Data represented as the mean ± s.d. of three independent experiments and were analyzed by two-way ANOVA with multiple comparisons, followed by Bonferroni post hoc test for significance. **p < 0.01, ****p < 0.0001 versus si-CTL, ####*p* < 0.0001 versus si-IRF3. **C** The BGC823 cells were transfected as described in B and starved for 12 h following by stimulation with *H. pylori* for further 48 h, the RLU was detected using dual‐luciferase reporter assay system (Promega). Data represented as the mean ± s.d. of three independent experiments and were analyzed by two-way ANOVA with multiple comparisons, followed by Bonferroni post hoc test for significance. ****p < 0.0001 versus si-CTL. **D** left panel: BGC823 cells were serum starved and treated as indicated for 1 h. The whole cell lysates were immunoprecipitated with an anti-IRF3 antibody to collect the chromosome segment that binding of IRF3 to KAT2 promoter in vivo, the fragments were amplified and quantified by real-time PCR. right panel: real-time PCR of a nonspecific region corresponding to CDS of the KAT2 gene enriched by IRF3 (negative control). Data represent the mean ± s.d. of three independent experiments and were analyzed by two-way ANOVA with multiple comparisons, followed by Bonferroni post hoc test for significance. ***p < 0.001. **E** BGC823 cells were starved for 12 h following by stimulation with or without *H. pylori* for 1 h, the ChIP was performed with anti-IRF3 antibody as described in D. Data represent the mean ± s.d. of three independent experiments and were analyzed by two-way ANOVA with multiple comparisons, followed by Bonferroni post hoc test for significance. ****p < 0.0001

Activation of Interferon Regulatory Factor 3 (IRF3) signaling has been shown to regulate type I interferon β (IFN- β) expression [43], which is belong to tryptophan cluster factors [44]. To explore whether *H. pylori*-dependent expression of KAT2 could be attributed to activation of IRF3 signaling. As shown in Fig. 3A, overexpression of IRF3 in BGC823 cells led to a significant increased relative luciferase unit (RLU) of KAT. While depletion of IRF3 expression in BGC823 cells drastically inhibited KAT2 transactivity, which further reversed the enhancement of KAT2 transactivity caused by CagA and/or VacA (Fig. 3B). In line with this, *H. pylori* infection in BGC823 cells led to increase KAT2 transactivation, while knockdown of IRF3 expression could reverse the enhanced KAT2 transactivation caused by *H. pylori* infection (Fig. 3C), these finding suggested that IRF3 is vital for *H. pylori-*mediated KAT2 expression.

To further confirm the possibility that upregulation of KAT2 transactivation resulting from CagA/VacA stimulation or *H. pylori* infection was attributed to the increased binding of IRF3 to the KAT2 promoter sequence, we utilized chromatin immunoprecipitation (ChIP) to confirm this hypothesis. As shown in Fig. 3D, the baseline of the binding of IRF3 to the KAT2 promoter region were dramatically increased in response to CagA/VacA treatment in BGC823 cells. As a negative control, no nonspecific amplifying was obtained using the primer targeted CDS of KAT2, indicating that the IP and real-time PCR-based amplification of the KAT2 promoter sequence was specific. In addition, *H. pylori* infection led to a significant binding of IRF3 to KAT2 promoter in BGC823 cells (Fig. 3E). Taken together, these results showed that IRF3 is critical for *H. pylori* -mediated KAT2 expression.

### *H. pylori* triggered cGAS/STING/IRF3 cascade signaling

**Fig. 4:**
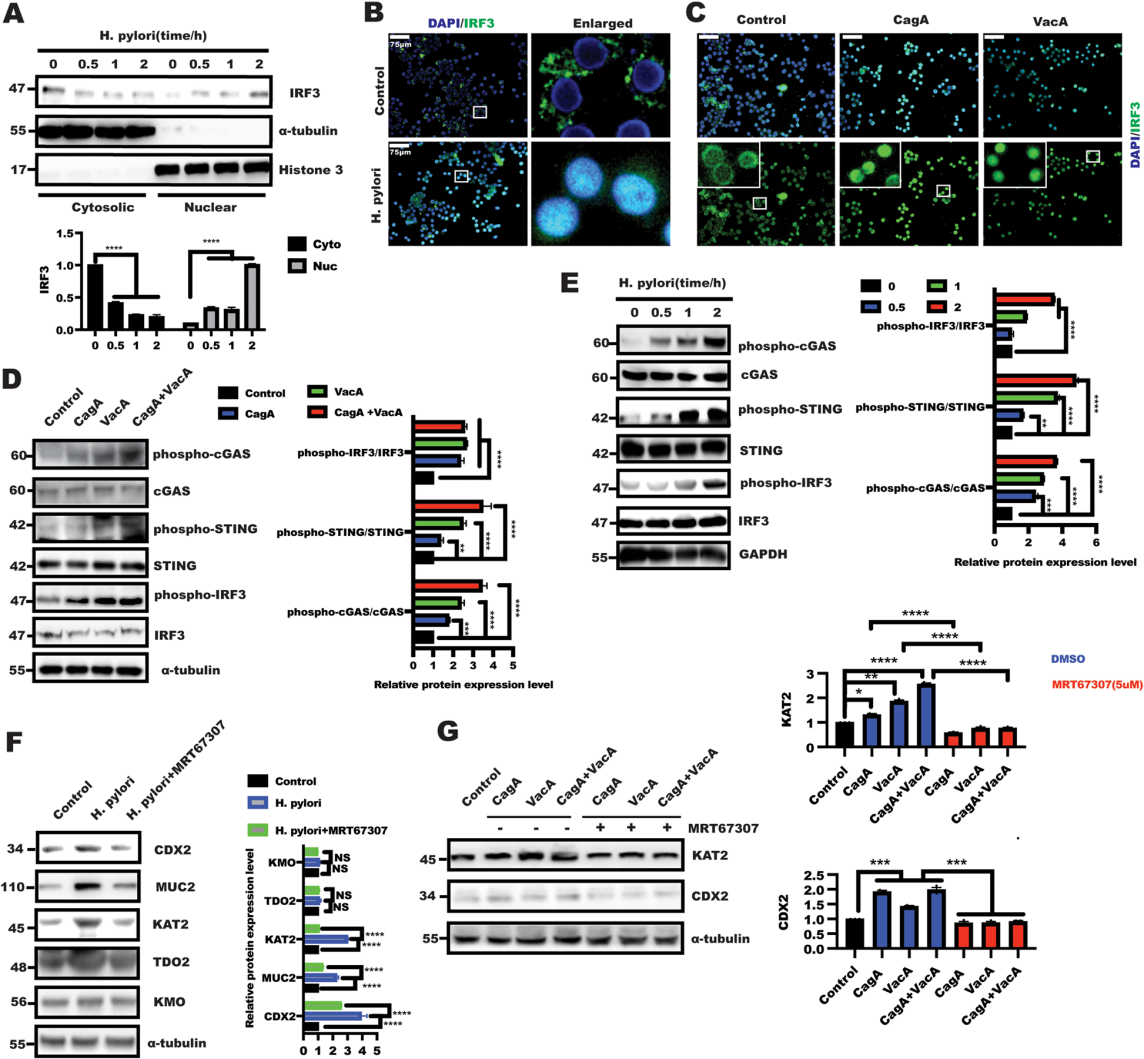
*H. pylori* mediated the cGAS-STING-IRF3 cascade pathway (**A**) BGC823 cells were serum starved for 24 h and then left untreated or treated with *H. pylori* for another 1 h, respectively. nuclear (Nuc) and cytosolic (Cyt) IRF3 were tested by immunoblotting. α-tubulin and Histone 3 were taken as internal controls for the cytosolic and nuclear fractions, respectively. **B** Immunofluorescence of IRF3 localization in BGC823 cells treated with or without *H. pylori* infection for 1 h after serum starved for 24 h. **C** After serum starvation for 24 h, BGC823 cells were treated with CagA, VacA and CagA + VacA for 1 h, respectively. Immunofluorescence was performed to display IRF3 nuclear translocation. After serum starvation for 24 h, BGC823 cells were treated with CagA, VacA and CagA + VacA (**D**) for 1 h or *H. pylori* (**E**) for indicated time. The total protein was collected to detect indicated protein with corresponding antibodies. Total cGAS/STING/IRF3 was determined to ensure equal loading. Data represent the mean ± s.d. of three independent experiments and were analyzed by one-way ANOVA with multiple comparisons, followed by Dunnett post hoc test for significance versus Control. **p < 0.01, ***p < 0.001, ****p < 0.0001. After serum starvation for 24 h, BGC823 cells were pre-treated for 1 h with IRF3 inhibitor MRT67307(5uM) and stimulated with *H. pylori* (**F**) or CagA, VacA and CagA + VacA (**G**) for additional 48 h. The total lysate was harvested to detect indicated proteins, Data represent the mean ± s.d. of three independent experiments and were analyzed by One-way ANOVA test for significance for F and two-way ANOVA with multiple comparisons, followed by Dunnett post hoc test for significance for G. *p < 0.01, ***p < 0.001, ****p < 0.0001

The above results suggested IRF3 was required for *H. pylori-*mediated KAT2 expression, the exact mechanism through which *H. pylori* regulated IRF3 signaling remained to be identified. The stimulator of interferon genes (STING)-IRF3 pathway has recently been shown to play an important role in immune and metabolic diseases. IRF3,a transcription factor, was activated by phosphorylation and translocated into the nucleus, initiating transcription of IFN-β and other antiviral genes [45]. As shown in Fig. 4A, a subcellular fractionation approach was utilized to analyze the influence of *H. pylori* on the level of nuclear IRF3 in BGC823 cells. The results showed that *H. pylori* infection led to a significant IRF3 nuclear translocation in the time course experiment (Fig. 4A). In line with this, immunofluorescence staining further confirmed that a remarkable nuclear translocation of IRF3 was observed in response to *H. pylori* infection *and* CagA/VacA stimulation in comparison with control group (Fig. 4B-C).

The above results indicated that *H. pylori* have an pivotal role in IRF3 nuclear translocation. Here, we sought to further reveal the possible mechanisms underlying IRF3 nuclear translocation caused by *H. pylori*. Because cGAS was activated in response to double-stranded DNA in a sequence-independent manner, and activation of cGAS-STING/IRF3 signaling is the classical pathway that mediates that mediated IRF3 nuclear translocation, which focused us to explore the effect of *H. pylori* on cGAS-STING/IRF3 signaling. WB analysis showed that in BGC823 cells, a significant upregulation of phosphorylation of cGAS/STING/IRF3 was observed in response to CagA and VacA stimulation for 1 h (Fig. 4D) and *H. pylori* infection for time course experiment (Fig. 4E). Moreover, inhibition of cGAS/STING/IRF3 signaling by MRT67307 in BGC823 cells could rescue the effect of *H. pylori* and its virulence factor on KAT2, even CDX2 and MUC2 expression (Fig. 4F-G). Taken together, these finding suggested *H. pylori* triggered cGAS/STING/IRF3 activation to induce IRF3 nuclear translocation, initiating KAT2 expression.

### IRF3 Inhibition protected against *H. pylori-*mediated gastric intestinal metaplasia In vivo

**Fig. 5:**
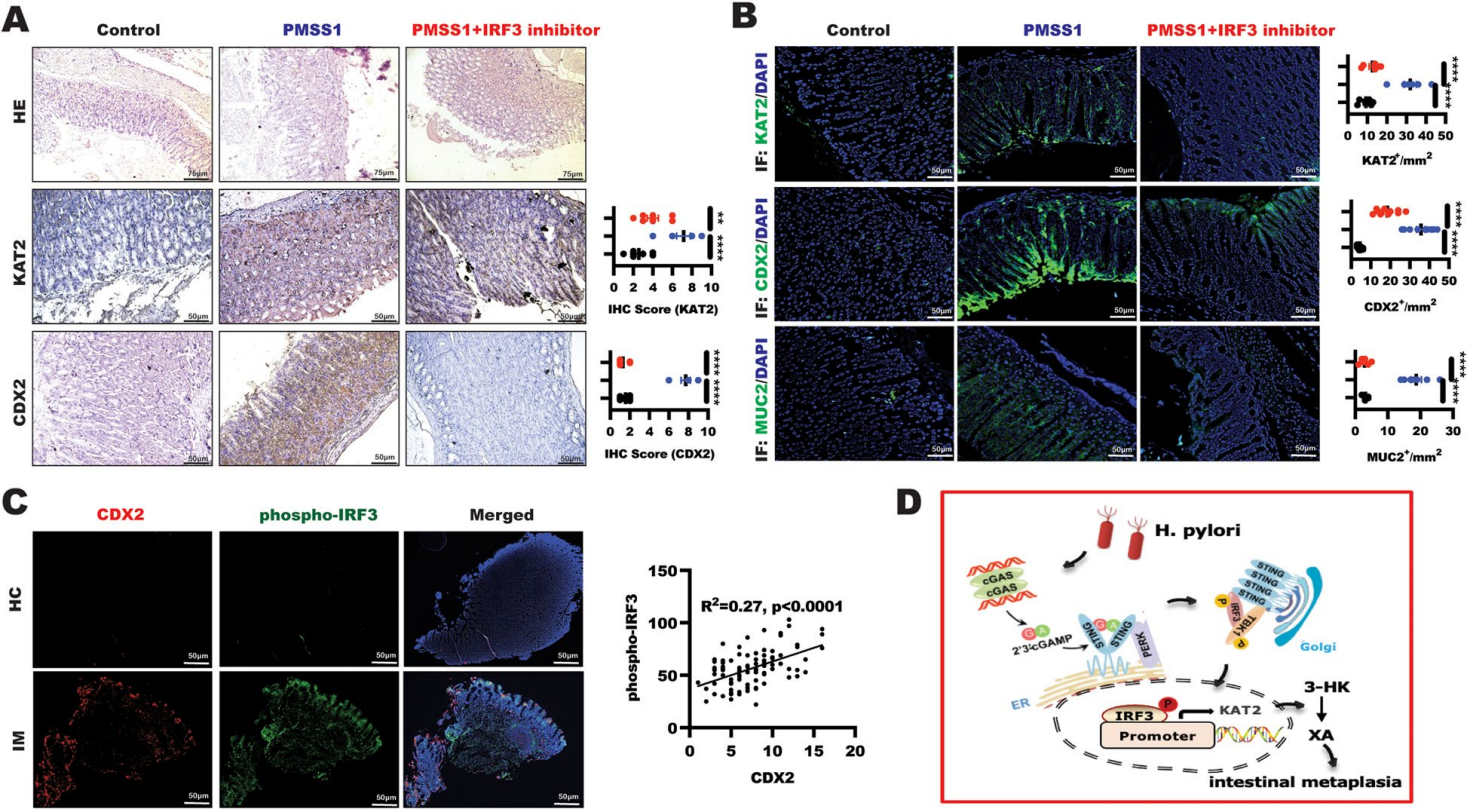
IRF3 inhibition alleviated H. pylori-infected gastric intestinal metaplasia. **A** HE staining of representative histological features of gastric mucosa of C57BL/6 mice in different groups (scale bars: 75 μm), IHC experiment staining showing representative images of CDX2 and KAT2 expression in gastric tissue from the indicated groups of mice (scale bars: 50 μm), Data represented the mean ± s.d. and was analyzed by One-way ANOVA test for significance. ****P < 0.0001; **P < 0.01. **B** Immunofluorescence was performed to detect KAT2, CDX2 and MUC2 expression in gastric mucosa tissue in indicated group, respectively. Data represented the mean ± s.d. and was analyzed by One-way ANOVA test for significance. ****P < 0.0001. **C** Immunofluorescence was performed to examine phosphor-IRF3 and CDX2 expression in gastric mucosa tissue in indicated group, respectively. The relationship of the indicated protein expression was analyzed using simple linear regression, ****P < 0.0001 (**D**) Schematic model of *H. pylori* triggered XA production to induced CDX2 in gastric epithelial cells. *H. pylori* triggered cGAS/STING/IRF3 pathway to promote IRF3 nuclear translocation in gastric epithelial cells, leading to increase the binding of IRF3 to KAT2 promoter and induce KAT2 transactivation, which further induced XA production and CDX2-mediated gastric intestinal metaplasia

To further provide insights into the effect of IRF3 inhibition on KAT2 expression in *H. pylori*-induced gastric intestinal metaplasia model. Mice were inoculated by oral gavage with *H. pylori* (PMSS1 strain) strain combined with IRF3 signal inhibitor MRT67307. The gastric tissues from uninfected or *H. pylori*-infected mice combined with or without MRT67307 treatment were subjected to immunohistochemical staining of CDX2 and MUC2. As shown in Fig. 5A, histological analysis of the gastric mucosa revealed that mice infected with the H. pylori PMSS1 strain showed significant inflammation, and both CDX2 and KAT2 staining were significantly observed in the *H. pylori*-infected mice, of note, these phenomena were alleviated after receiving MRT67307 treatment. What’s more, the analysis of IF staining displayed that gastric intestinal metaplasia characterized by enhanced CDX2 and MUC2 expression was drastically suppressed in the mucosa in *H. pylori*-infected mice after MRT67307 treatment, which was attributed to the decreased KAT2 expression caused by IRF3 inhibitor MRT67307 (Fig. 5B). This in vivo experiment demonstrated that IRF3 is a critical regulator of *H. pylori*-induced KAT2-mediated kynurenine pathway activation to trigger intestinal metaplasia. The strategy that targeting IRF3 could protect against the progression of H. pylori-induced gastric intestinal metaplasia.

The above results suggested the critical role of KAT2 in H. pylori-mediated gastric intestinal metaplasia, which focused us to further explore the clinical relationship between KAT2 and CDX2. As shown in Table 1, we have recruited 26 subjects, including 14 *H. pylori*-infected patients diagnosed with gastric intestinal metaplasia, 12 healthy controls with aged between 30 and 70 years. Detailed clinical characteristics of the subjects, which are not public, could be available upon reasonable request. IF was performed in a set slide tissue to analyze phosphorylated IRF3 and CDX2 expression in gastric intestinal metaplasia with *H. pylori* infection. The results showed that phosphorylation of IRF3 was significantly increased in the gastric epithelial cells in patients with *H. pylori*-mediated gastric intestinal metaplasia characterized by enhanced CDX2 expression in IM group in comparsion to HC group (Fig. 5C), further linear regression analysis showed that phosphorylation of IRF3 is positively correlation with CDX2 (*p* < 0.0001). Overall, these results suggested that activation of IRF3 pathway is critical role for *H. pylori*-infected gastric intestinal metaplasia.

**Table 1:** The characteristic of subjects enrolled in this study

Vaiable	Number of patients (%)	
Total	Gastric intestinal metaplasia	Healthy control
Age	14(53.8%)	12(46.2%)
< 45	3(11.5%)	1(2.9%)
> = 45	11(42.3%)	11(42.3%)
Gender		
Female	4(28.6%)	5(19.2%)
Male	10(38.5%)	7(13.7%)
Stage		
+ +	8(30.8%)	-
+ + +	6(23.0%)	-

## Discussion

Gastric intestinal metaplasia (IM), a precancerous or para-cancerous lesion, is strongly associated with chronic *H. pylori* infection. In this study, as shown in Fig. 5D, we revealed that *H. pylori* contributed to IM by triggering KAT2-mediated kynurenine pathway through IRF3 pathway. KAT2 inhibition in gastric epithelial cells could reverse the promotion of *H. pylori* on CDX2 expression. Mechanically, *H. pylori* activated cGAS/STING/TBK1/IRF3 signaling, resulting in nuclear translocation of IRF3 and initiating KAT2 transactivation, which further induced XA production to induce IM characterized by enhanced CDX2 expression. Inhibition of IRF3 could rescue the expression of KAT2 caused by *H. pylori*. Most importantly phosphorylation IRF3 was positively correlation with CDX2 expression in clinical sample with *H. pylori* infection diagnosed with IM, suggesting IRF3-induced kynurenine pathway is critical for *H. pylori*-mediated IM. Taken together, these finding greatly extended our insight into the metabolic reprogramming of kynurenine pathway in *H. pylori*-induced IM.

Up to date, there is limit available reports about the metabolic reprogramming during *H. pylori*-induced IM. Cellular metabolism regulates normal cell functions as well as the pathophysiology of multiple disease conditions. Recently, 11 metabolites involved in glycolysis, tricarboxylic acid (TCA) cycle, and amino acid metabolism, such as asparagine, serine, glycine, citric acid, malic acid and isocitric acid, were found to be significantly altered in AGS cells infected with *H.pylori *[46]. In this work, we have identified XA was largely enhanced in gastric epithelial cells in response to CagA and VacA or *H. pylori* treatment, and further analysis showed that XA treatment in gastric epithelial cells could induce CDX2 expression, leading to gastric IM, which was attributed to enhanced KAT2 expression confirmed by qPCR, WB. Interestingly, the study demonstrated that XA-stimulated cGMP synthesis through GEP1 (gametogenesis essential protein 1) and the subsequent signaling and cellular events, such as Ca^2+^ mobilization, gamete formation, and gametes egress out of erythrocytes [47, 48]. However, the further work is required to discuss the work that how XA induced CDX2 expression and the possible function of XA in *H. pylori*-induced pathology, including *H.pylori* replication and colonization, immunity, and drug resistance. In addition to XA, the investigation is required to explore that there is any possible metabolites in kynurenine pathway involved in *H. pylori*-induced IM, such as kynurenine, 3-hydroxy kynurenine.

In line with the Song et al. study showed that infection with *H. pylori* is well recognized as a kind of genotoxic DNA pathogens to trigger STING signaling, including IRF3 phosphorylation [49], we found that phosphorylation and nuclear translocation of IRF3 was increased in gastric epithelial cells in response to *H. pylori* stimulation, which was attributed to activation of cGAS-STING pathway, including phosphorylation of TBK and STING. Further analysis suggested that *H. pylori* infection in gastric epithelial cells led to the binding of IRF3 to KAT2 promoter confirmed by ChIP and luciferase assay, identifying KAT2 is a direct target of IRF3. What’s more, inhibition of IRF3 in gastric epithelial cells could reverse the enhanced KAT2, even CDX2 and MUC2 expression caused by *H. pylori*, while KAT2 suppression by could rescue the inducible of CDX2 expression in gastric epithelial cells in response to *H. pylori* stimulation. However, in addition to KAT2, the critical molecular through which *H. pylori*triggered IM could be required to address, and the work is also needed to explore the novel pathway involved in *H. pylori*-mediated kynurenine pathway. This unsolved issue would be addressed in our future work.

In summary, this work is the first to unravel the role and mechanism that *H. pylori* induced gastric intestinal metaplasia by activation of kynurenine pathway in gastric epithelial cells. *H. pylori* promoted kynurenine pathway and XA production through activation of IRF3, which further led to CDX2 and MUC2 expression in gastric epithelial cells. We have identified KAT2 is a direct target of IRF3, and most importantly, *H. pylori* regulated IRF3 phosphorylation, leading to IRF3 nuclear translocation and increase the binding of IRF3 to KAT2 promoter, which further enhanced KAT2 transcription. Despite how XA induced CDX2 expression remained unknown, the current study provided insights into the novel metabolic reprogramming mechanism *of H. pylori* in gastric intestinal metaplasia.

## Conclusions

This work has revealed *H. pylori* triggered cGAS/STING/IRF3-mediated kynurenine pathway of tryptophan metabolism to promote XA production, which further induced gastric intestinal metaplasia.

## Supplementary Information

**Additional file 1. **Thedetailed information of subjects enrolled in the study.

## Data Availability

The datasets generated during and/or analyses during the current study are available from the corresponding author on reasonable request.

## Abbreviations

3HK: 3-Hydroxy kynurenine
3HAA: 3-Hydroxy anthranilic acid
ACVR1: Activin A receptor type I
ACMSD: 7, 2-Amino-3-carboxymuconic acid semialdehyde decarboxylase
ALKBH5: Alkylation repair homolog protein 5
BCL2: B-cell lymphoma-2
BA: Bile acids
CagA: Cytotoxin associated gene A
CDX2: Caudal-related homeobox transcription factor-2
CRC: Colorectal cancer
ChIP: Chromatin Immunoprecipitation
COL12A1: Collagen Type XII
DMEM: Dulbecco's Modified Eagle Medium
DNL: De novo lipogenesis
FBS: Fetal bovine serum
FXR: Farnesoid X Receptor
FASN: Fatty acid synthase
GC: Gastric cancer
GECs: Gastric epithelial cells
HK2: Hexokinase 2
H. pylori: Helicobacter pylori
HNF4α: Hepatocyte nuclear factor 4 alpha
LC–MS: Liquid chromatography-mass spectrometry
IRF3: Interferon regulatory factor 3
IF: Immunofluorescence
IM: Intestinal metaplasia
IL: Interleukin
KYNA: Kynurenine to Kynurenic acid
KYNU: Kynureninase
KAT: Kynurenine aminotransferase
KMO: Kynurenine 3-monoxygenase
KYN: Kynurenine
IDO1: Indoleamine-2,3-dioxygenase-1
NF-κB: Nuclear factor kappa-light-chain-enhancer of activated B cells
Trp: Tryptophan
TDO2: Tryptamin 2,3 dioxygenase
PBS: Phosphate Buffer solution
PIC: Picolinic acid
QA: Anthranilic acid
QUIN: Quinolinic acid
QPRT: 3-Hydroxyanthranilate-3,4-dioxygenase
QPCR: Quantitative polymerase chain reaction
RLU: Relative luciferase unit
SHMT2: Serine hydroxymethyltransferase-2
STAT3: Signal transducer and activator of transcription 3
SOX2: SRY (sex determining region Y)-box 2
SDS-PAGE: Sodium dodecyl sulfate polyacrylamide gel electrophoresis
TBST: Tris Buffered Saline Tween-20
VacA: Vacuolating cytotoxin A
WB: Western blotting
XA: Xanthurenic acid
